# Shotgun Proteomics Analysis, Functional Networks, and Peptide Biomarkers for Seafood-Originating Biogenic-Amine-Producing Bacteria

**DOI:** 10.3390/ijms24097704

**Published:** 2023-04-22

**Authors:** Ana González Abril, Pilar Calo-Mata, Karola Böhme, Tomás G. Villa, Jorge Barros-Velázquez, Manuel Pazos, Mónica Carrera

**Affiliations:** 1 Department of Food Technology, Spanish National Research Council (CSIC), Institute of Marine Research (IIM-CSIC), 36208 Vigo, Spain; 2 Department of Microbiology and Parasitology, Faculty of Pharmacy, University of Santiago de Compostela, 15898 Santiago de Compostela, Spain; 3 Department of Analytical Chemistry, Nutrition and Food Science, Food Technology Division, School of Veterinary Sciences, University of Santiago de Compostela, Campus Lugo, 27002 Lugo, Spain

**Keywords:** biogenic-amine-producing bacteria, shotgun proteomics, mass spectrometry, (LC-ESI-MS/MS), food, seafood, bacterial identification

## Abstract

Biogenic amine-producing bacteria are responsible for the production of basic nitrogenous compounds (histamine, cadaverine, tyramine, and putrescine) following the spoilage of food due to microorganisms. In this study, we adopted a shotgun proteomics strategy to characterize 15 foodborne strains of biogenic-amine-producing bacteria. A total of 10,673 peptide spectrum matches belonging to 4081 peptides and corresponding to 1811 proteins were identified. Relevant functional pathways were determined, and strains were differentiated into hierarchical clusters. An expected protein-protein interaction network was created (260 nodes/1973 interactions). Most of the determined proteins were associated with networks/pathways of energy, putrescine metabolism, and host-virus interaction. Additionally, 556 peptides were identified as virulence factors. Moreover, 77 species-specific peptide biomarkers corresponding to 64 different proteins were proposed to identify 10 bacterial species. This represents a major proteomic dataset of biogenic-amine-producing strains. These results may also be suitable for new treatments for food intoxication and for tracking microbial sources in foodstuffs.

## 1. Introduction

**Figure 1:**
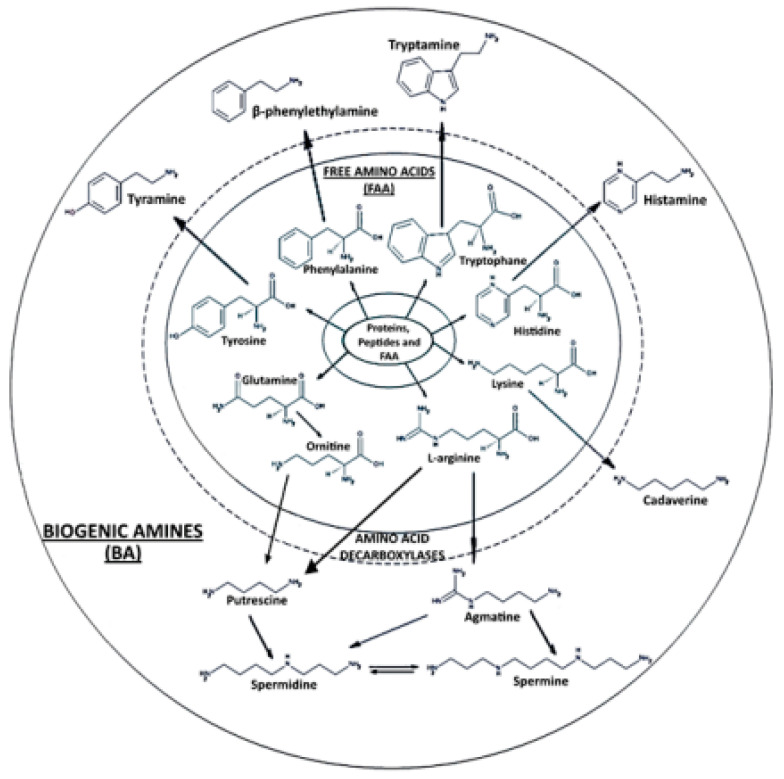
Biogenic amines. (Modified from [4]; common license.)

Biogenic amines (BAs) are low-molecular-weight nitrogenous compounds that are principally generated by the decarboxylation of free amino acids or by the deamination/amination or transamination of aldehydes and ketones [1]. In food, BAs are created in the process of microbial, animal, and vegetable metabolism since the primary source of BAs is the decarboxylation of amino acids by fermentation, putrefaction, or decomposition [2]. Fish, cheese, soy sauce, meat, wine, and beer are some products that often generate BAs [3]. Histamine, cadaverine, tyramine, putrescine, spermidine, and spermine are the main BAs used as indicators for food spoilage [4] (Figure 1).

Histamine is the most common BA responsible for food poisoning. Histamine was originally described by Dale in 1910 [5] as a small molecule produced by the decarboxylation of histidine [5]. Histamine induces a variety of biological processes, including the regulation of physiological functions in the gut, the stimulation of the nasal mucous membrane, and the release of gastric acids; additionally, the more serious processes it induces involve vasodilation and inflammation for triggering anaphylactic responses, which are similar to allergic responses and can be life-threatening [6]. These BAs can be degraded by two enzymes, namely, diamine oxidase or histaminase and histamine-N-methyltransferase; some point mutations in the genes encoding these enzymes are associated with several disorders, such as ulcerative colitis and even autism. This suggests that rapid histamine removal is important to prevent harmful pathological events such as a bronchospasm, a dangerous symptom occurring in anaphylactic reactions.

Although putrescine and cadaverine are also common BAs present in foods, these compounds were believed to only be toxic in large concentrations. However, in 2019, del Rio et al. [7] carried out an in vitro study demonstrating that these two BAs display cytotoxic action (causing cell necrosis) at concentrations found in some foodstuffs (such as fish and fermented food).

In humans, in addition to endogenously produced histamine and trace amines derived from commensal bacteria in the gut, BAs can be internalized through the ingestion of food. There are a variety of bacteria that synthetize and secrete histamine and other BAs as metabolic products, thus generating significant amounts of these compounds that can accumulate in foodstuffs (as a result of improper storage). In 1999, Ben-Gigirey et al. [8,9] reported the isolation of both cadaverine- and histamine-producing bacteria from frozen or fresh albacore (*Thunnus alalunga*). BAs can accumulate in food via the metabolic processes of microorganisms that produce decarboxylases; these enzymes can exert their action on amino acid precursors, which is an absent process in the ‘normal’ metabolism of animals or plants. If a bioactive amine is produced in large quantities, the foodstuffs involved are prime candidates for food poisoning and could constitute a major threat to public health due to severe symptoms of intoxication. On the other hand, even low BA levels can lead to food intolerance among susceptible people, particularly those afflicted with low levels of diamine oxidase activity, which could be exacerbated by the intake of histamine-containing foods. An example is the so-called ‘scombroid food poisoning’, one of the main forms of seafood poisoning; this poisoning results from eating fish containing histamine (scombrotoxin), which is produced by contaminating bacteria. The symptoms appear soon after fish consumption and include headaches, flushed skin, itchy skin, or abdominal cramps and can last for 2 to 3 days. Depending on the geographical zone, different types of fish can be responsible for food poisoning, including bluefish, tuna, sardines, anchovies, and turbot [10]; these fish contain high levels of histidine, which is rapidly transformed into histamine by bacteria during storage [11,12]. Fermented foods can also contain high levels of BAs, which are undoubtedly produced by contaminating microorganisms during fermentation that is improperly controlled [13]. Hence, it is essential to identify the critical step in fermentation that results in bacterial contamination; this is particularly important in the dairy industry, as a variety of products are produced by microbial fermentation, such as cheeses ripened with bacterial or yeast starters, including lactic acid bacteria. It is very concerning that high amounts of BAs were not only detected in yogurt but also in both raw and processed milk, including pasteurized, UHT, and reconstituted powered milks [1,14].

Contaminative biogenic-amine-producing bacteria usually belong to the group of ‘normal’ microbiota that inhabit animals or plants from which food originates, and these microorganisms include members of the family Enterobacteriaceae (i.e., *Escherichia coli*, *Klebsiella* spp., *Hafnia alvei*, *Proteus* spp., *Salmonella* spp., and *Serratia* spp.), the family Vibrionaceae (i.e., *Vibrio alginoliticus*), and *Pseudomonas* or *Pseudomonas*-like species. Considering that these bacteria are usually present in the starting material and that most microorganisms can grow extremely fast, it is advisable to promptly commence the food preservation process and quickly and unambiguously identify the relevant microbial organisms present in foodstuffs. Takahashi et al. (2003) [15] established a PCR-based strategy for the quick determination of histamine-producing Gram-negative bacteria, while Coton and Coton (2005) [16] applied a similar method (multiplex PCR) for the discovery of bacterial histidine decarboxylase (*hdc*) genes present in Gram-positive bacteria (*Lactococcus*, *Enterococcus*, and *Streptococcus*), which have been described as more significant producers of BAs in fermented food [17,18,19]. Real-time PCR was also utilized for the quantification of histamine in wine [20], cheese products [21], and fish [3]. More recently, new techniques involving LC-ESI-MS/MS-based proteomics have provided a rapid approach to identifying the bacterial species comprising and the bacteriophages present in pathogenic bacteria [22,23,24,25]. This approach is also valid for studying the different antibiotic resistance mechanisms displayed by bacteria, such as the strategies used by pathogenic streptococcal species [26] and *Listeria monocytogenes* [27]. Another advantage of this novel method is that its corresponding analyses can be directly obtained from foodstuffs, as they do not require bacterial enrichment; hence, the microorganisms being studied do not have to be cultivated in a laboratory. There are currently a variety of techniques that can be applied to quantitate the levels of biogenic amines secreted by actively growing BA-producing bacteria, including HPLC-based methods [28] or classic microbiological procedures such as the approach taken by Tao et al. (2009) [29], which involves bacterial growth in differential agar media.

In this manuscript, the most relevant BAs in seafoods (fish) are addressed, and the relevance of these molecules for food quality and safety are reported. Fish is an extremely perishable food product and contains a vulnerable matrix that can include high levels of BAs [1]. In this work, we used a shotgun proteomic technique to quickly and easily characterize 15 different foodborne strains of biogenic-amine-producing bacteria for the first time. The proteome repository was then subjected to some functional bioinformatics examinations, such as (i) functional pathway, gene ontology (GO), and hierarchical clustering analyses; (ii) protein network analysis; (iii) the identification of virulence factors; and (iv) the selection of putative species-specific peptide biomarkers for the distinction of foodborne biogenic-amine-producing bacteria.

## 2. Results and Discussion

### 2.1. Shotgun Proteomics Data Repository

Fifteen different seafood-based biogenic-amine-producing bacteria were analyzed in this study (Table 1). Bacterial peptides were obtained via the trypsin digestion of protein mixtures and a subsequent analysis using LC-ESI-MS/MS, as presented previously [22,23,24,30]. A total of 10,673 peptide spectrum matches (PSMs) belonging to 4081 nonredundant peptides were determined, which belonged to 1811 annotated proteins from the Proteobacteria UniProt/TrEMBL database (August 2022) (Supplementary Data S1). The MS/MS proteomics data were deposited in the ProteomeXchange Consortium via the PRIDE [31] storage website with the dataset identifier PXD039320.

To the best of our knowledge, the current data constitute the largest dataset of proteins and peptides of seafood-based biogenic-amine-producing bacteria identified to date. This valuable protein repository will add novel and important content to public protein databases and will hopefully be useful for novel research.

### 2.2. Label-Free Quantification (LFQ) of Biogenic-Amine-Producing Bacteria and Hierarchical Clustering

The relative label-free quantification of each type of bacteria was also executed to define the level of protein abundance in each sample. Supplementary Data S2 contains these results.

**Figure 2:**
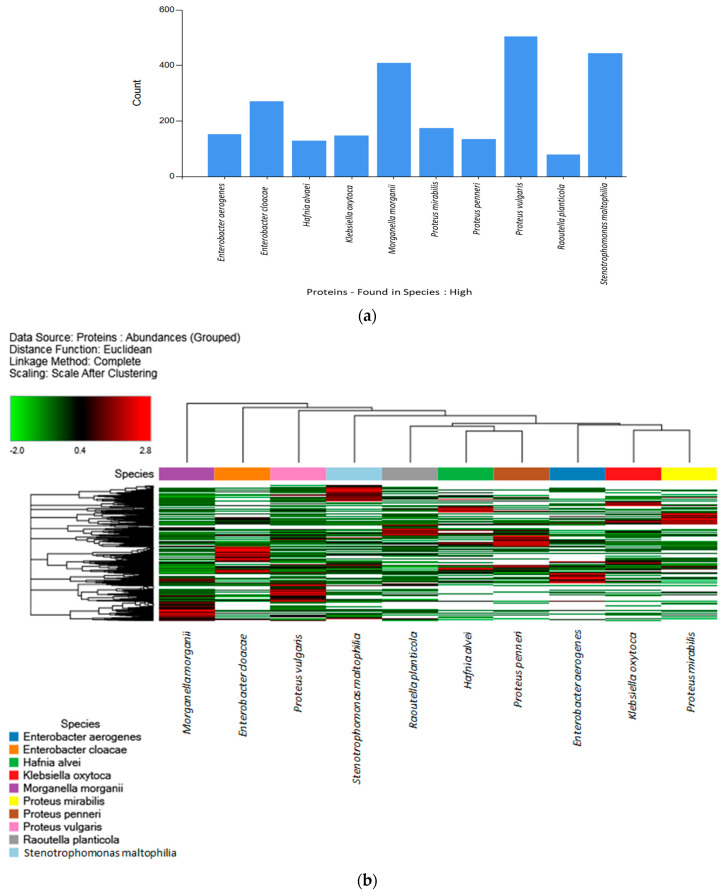
(**a**) Distribution of the high-abundance proteins for each biogenic-amine-producing strain determined via LFQ; y-axis (count) is the number of identified proteins. (**b**) Heatmap from the shotgun proteomics analysis of 15 different biogenic amine foodborne strains. Every bar corresponds to the presence or absence of a particular protein. Red = upregulated proteins; green = downregulated proteins. Euclidean hierarchical distances were sorted for all strains. Three principal clusters were differentiated.

Comparisons of the high-abundance proteins of each species and strain were performed. Figure 2a displays the distribution of the high-abundance proteins determined for each of the 15 strains. Among them, *Proteus vulgaris*, *Stenotrophomonas maltophilia*, and *Morganella morganii* were the three main species with the most high-abundance proteins. The distribution of the high-abundance proteins for all samples analyzed via LFQ is illustrated in a heatmap diagram in Figure 2b. Euclidean hierarchical distance was used to differentiate three main clusters. Cluster A (strains H6, H2, H9, and H14: *Morganella morganii*, *Enterobacter cloacae*, *Proteus vulgaris*, and *Stenotrophomonas maltophilia*), Cluster B (strains H12, H3, and H8: *Raoutella planticola*, *Hafnia alvei*, and *Proteus penneri*), and Cluster C (strains H1, H4, and H7: *Enterobacter aerogenes*, *Klebsiella oxytoca*, and *Proteus mirabilis*). As in Figure 2a, the clusters of Figure 2b were divided according to the number of proteins that were more upregulated (Red) (as determined via LFQ) versus those proteins that were more downregulated (Green) for the different strains.

**Figure 3:**
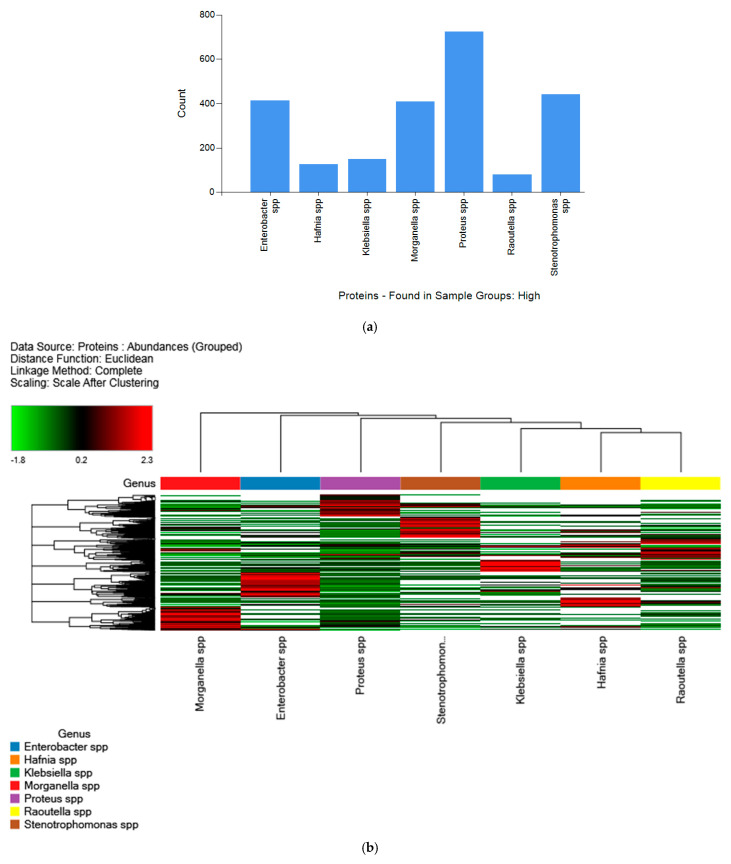
(**a**) Distribution of the high-abundance proteins for each genus determined using LFQ; (**b**) Heatmap from the shotgun proteomics analysis of 15 different biogenic amine foodborne strains according to different genera. The y-axis (count) represents the number of identified proteins. Every bar corresponds to the presence or absence of a particular protein. Red = upregulated proteins; green = down regulated proteins. The Euclidean hierarchical distances were sorted according to different genera (*Enterobacter* spp., *Hafnia* spp., *Klebsiella* spp., *Morganella* spp., *Proteus* spp., *Raoultella* spp., and *Stenotrophomonas* spp. Seven principal clusters were differentiated, which corresponded to the different genus types.

Regarding the different genera, Figure 3a shows the high-abundance proteins for each genus (*Enterobacter* spp., *Hafnia* spp., *Klebsiella* spp., *Morganella* spp., *Proteus* spp., *Raoultella* spp., and *Stenotrophomonas* spp.). Among them, *Proteus* spp. was the most represented genus with the most high-abundance proteins. The distribution of the high-abundance proteins for all samples grouped by genus and analyzed via LFQ is illustrated in a heatmap diagram in Figure 3b. Finally, all strains were arranged according to Euclidean hierarchical distance. Seven principal clusters were differentiated, which corresponded to the different genus types.

To obtain further insights regarding functional interpretation, the present repository was investigated using several functional in silico analyses, comprising (i) functional pathways, GO enrichment and hierarchical clustering, (ii) functional network analysis, (iii) the discovery of virulence factors, and (iv) the selection of potential species-specific peptide biomarkers.

### 2.3. Functional Pathways and Gene Ontology (GO)

The global protein repository of foodborne strains of biogenic-amine-producing bacteria was individually examined using functional bioinformatics tools, such as functional pathway analysis and GO term enrichment.

**Figure 4:**
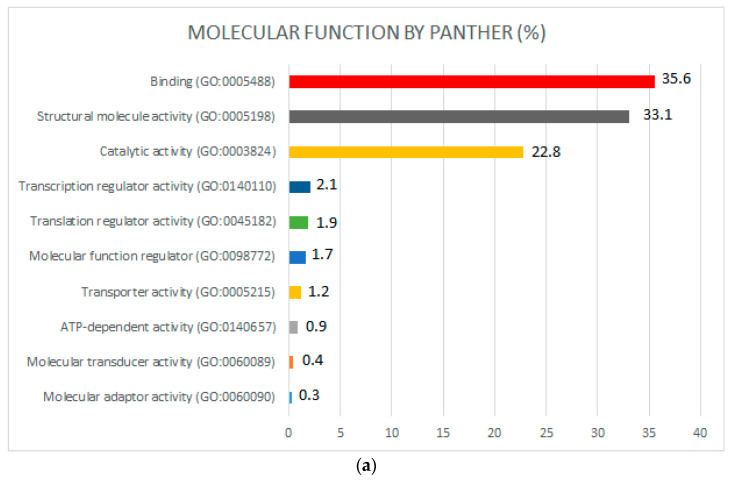

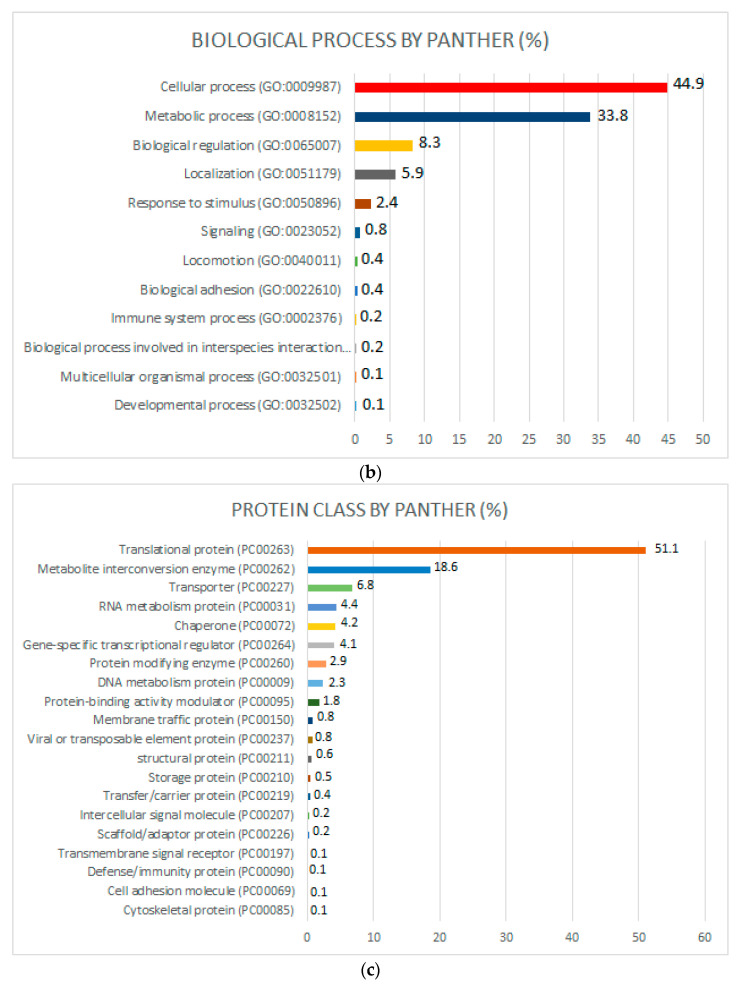
(**a**) Molecular functions of the biogenic-amine-producing bacterial proteome identified using shotgun proteomics and categorized via PANTHER using the gene names as inputs for the software; (**b**) biological processes of biogenic-amine-producing bacterial proteome identified via shotgun proteomics and categorized via PANTHER; (**c**) protein classes of biogenic-amine-producing bacterial proteome identified via shotgun proteomics and categorized via PANTHER.

PANTHER analysis was performed using gene names (considering all nonredundant proteins), revealing the presence of 10 different molecular functions (Figure 4a), 12 different biological processes (Figure 4b), and 20 different protein classes (Figure 4c) in the complete global proteome repository.

According to the molecular function classification procedure (Figure 4a), the most important molecular functions were binding (35.6%), structural molecule activity (33.1%), and catalytic activity (22.8%). Within the binding function group, ribosomal proteins, oxidorreductases, chaperones, DNA metabolism proteins, deaminases, isomerases, transferases, translation elongation factor proteins, mutases, and protein kinases were found. In the structural molecule activity group, ribosomal proteins and tubulins were detected. Regarding catalytic activity, decarboxylases, nucleotide kinases, oxidases, kinases, pyrophosphatases, isomerases, transferases, deaminases, proteases, dehydrogenases, and mutases were observed.

According to the classification of biological processes (Figure 4b), the most remarkable categories were cellular processes (44.9%), metabolic processes (33.8%), biological regulation (8.3%), localization (5.9%), response to stimulus (2.4%), and signaling (0.8%). Regarding cellular processes, ribosomal proteins, decarboxylases, pyrophosphatases, vesicle coat proteins, isomerases, transferases, and translocation initiation factors were found. Concerning the metabolic process group, ribosomal proteins, decarboxylases, pyrophosphatases, translation release factor, chaperone, isomerases, transferases, and metalloproteases were detected. In the biological regulation group, ribosomal protein, chaperone, membrane traffic protein, primary active transporter, and storage proteins were observed.

According to the classification of protein classes (Figure 4c), the most prominent classes were translational proteins (51.1%), metabolite interconversion enzymes (18.6%), and transporters (6.8%). Within the translational protein group, ribosomal protein and translation initiation/elongation/release factors were observed. In the metabolite interconversion enzyme group, different enzyme groups were observed, including dehydrogenases, carbohydrate kinases, aldolases, isomerases, hydrolases, glycosidases, transferases, oxidases, glucosidases, peroxidases, mutases, dehydratases, phospholiases, isomerases, and deaminases. Within the transporter category, ATP synthase, ATP-binding cassettes, amino acid transporters, and ion channels were detected.

The existence of high concentrations of decarboxylases in these functional classifications influences the formation of biogenic amines by the bacteria. During the deterioration of fish, the occurrence of bacterial strains with high proteolytic enzyme activity increases the breakdown of proteins as well as the accessibility of small peptides and specific free amino acids that are decarboxylated in particular biogenic amines [32]. In fish, the principal studied biogenic amines include histamine (derived from histidine), putrescine (derived from arginine, glutamine, methionine, and ornithine), cadaverine (derived from lysine), tyramine (derived from tyrosine), spermidine (derived from agmatine, methionine, putrescine, and spermine), and spermine (derived from agmatine, methionine, putrescine, and spermidine) [33].

### 2.4. Biogenic Amine-Related Proteins and Peptides Detected via LC-ESI-MS/MS

Table 2 summarizes the list of biogenic amine-related proteins and peptides detected via LC-ESI-MS/MS for the corresponding strains.

Agmatine and cadaverine are aliphatic polyamine biogenic amines derived from the amino acids arginine and lysine, respectively [33]. Two different related proteins (arginine ABC transporter substrate-binding protein and lysine–arginine–ornithine-binding periplasmic protein) and three different peptides (IDAVFGDTAVVTEWLK, C*TWVGSDFDSLIPSLK, and IGTDATYAPFSSK) were detected via shotgun proteomics in the *K. oxytoca* strain. The metabolism of agmatine and cadaverine requires the initial presence and transport of arginine or lysine, respectively, in the periplasm of the cells. In Gram-negative bacteria, solute-binding proteins are localized in the periplasm and involved in nitrogen compound transport (GO:0071705) and amine transport (GO:0015837).

Histamine is a heterocyclic biogenic amine derived from the amino acid histidine [2]. Histamine is present in most foods but is more abundant in fish and fishery products. This biogenic amine is the major agent behind “scombroid poisoning” or “histamine poisoning” [11,34]. A total of five different related proteins (histidine kinase, histidine phosphatase, histidine-binding periplasmic protein, histidine triad nucleotide-binding protein, and histidine ammonia-lyase) were detected via shotgun proteomics (Table 2). Nine peptides of histidine kinase (GO:0004673) were identified via LC-ESI-MS/MS analysis in different strains (IDSEDLPHVRASVAR (present in *M. morganii*), LAM*NLRTRLFLSISALITVALLGLLLGLVSVM*QM*AGSQ-EILIR (*S. maltophilia*), M*IAEAANADSKQAQR (*K. oxytoca* and *R. planticola*), TIDQINQQKIQLEQEIADRK (*P. penneri* and *P. vulgaris*), GEADATLDSEVSAWRAVAR (*P. vulgaris*), LSSELWNC*KIDPTQAEM*AM*INILANAR (*P. mirabilis*), SEASENTVDLIVEDEGSGIPK (*P. mirabilis*), NEEARDNLISELTAR (*P. vulgaris*), and RYAYSEQLGDLLQR (*S. maltophilia*)). In addition, a peptide from histidine phosphatase (GO:0101006) was detected via LC-MS/MS (HAQASEYGSALFVAVGQAKQVK) in the *H. alvei* strain. It is well known that histidine kinase/phosphatase regulates histamine synthesis and signal transduction by activating histidine decarboxylase through phosphorylation/dephosphorylation [35]. Moreover, a peptide of histidine-binding periplasmic protein (IGVLQGTTQETYGNEHWAPK) was detected in the *K. oxytoca* strain, and two peptides of histidine triad nucleotide-binding protein (EIPSDIVYQDELVTAFR, IAEQEGIAEDGYR) were detected in the *E. cloacae* strain. These proteins are involved in nitrogen compound transport and amine transport (GO:0071705, GO:0015837). Finally, a peptide (LAAM*QQALGAQIAAVEEDR) of histidine ammonia-lyase was identified via LC-ESI-MS/MS in the *M. morganii* strain. This cytosolic enzyme catalyzes the first reaction in histidine catabolism: the nonoxidative deamination of histidine to trans-urocanic acid (GO:0004397).

Putrescine is an aliphatic biogenic amine derived from the amino acids arginine or ornithine in one step or two steps after glutamine or methionine is transformed into ornithine and then putrescine. The ingestion of food containing high amounts of putrescine can lead to grave toxicological consequences. In fact, putrescine can react with nitrite to form N-nitrosamines, which are carcinogenic agents [36]. Additionally, putrescine induces significant effects that enhance the toxicological effects of other BAs, particularly histamine and tyramine [37]. In seafood such as fish, squid, and octopus, putrescines are also dominant biogenic amines [38]. Lysine–arginine–ornithine-binding periplasmic protein and two peptides (C*TWVGSDFDSLIPSLK; IGTDATYAPFSSK) were also identified via LC-ESI-MS/MS analysis in the *K. oxytoca* strain. The metabolism of putrescine also requires the initial presence and transport of arginine or ornithine in the periplasm of the cells (GO:0071705 and GO:0015837). In addition, three proteins responsible for glutamine and methionine transport and amino/amido transferase were identified via shotgun proteomics in *K. oxytoca* and *H. alvei* strains. These corresponded to glutamine ABC transporter periplasmic protein (peptide: AVGDSIEAQQYGIAFPK) present in *K. oxytoca*, glutamine-fructose-6-phosphate aminotransferase (IDAAQEAELIKALFEAPR) present in *K. oxytoca*, N-acetylglutaminylglutamine amidotransferase (SGANAAVDKALRLDSTVM*LVDDPVK) present in *H. alvei*, type 1 glutamine amidotransferase domain-containing protein (IFRTLALM*LLVTSATAFAASK) present in *P. mirabilis*, and L-glutamine-binding protein (ADAVIHDTPNILYFIK, AVGDSLEAQQYGIAFPK) present in *K. oxytoca* and *H. alvei* strains. Finally, the S-adenosylmethionine decarboxylase proenzyme (AdoMetDC) (ALSFNIYDVC*YAR) was detected in the *S. maltophilia* strain. It is involved in the synthesis of biogenic amines in several species that use aminopropyltransferases for this pathway. AdoMetDC is involved in the production of S-adenosyl-1-(methylthio)-3-propylamine (decarboxylated S-adenosylmethionine) [39]. In contrast to many amino acid decarboxylases that use pyridoxal 5′-phosphate as a cofactor, AdoMetDC uses a covalently bound pyruvate residue. This decarboxylase is involved in the polyamine biosynthetic pathway, as it generates the n-propylamine residue needed for the synthesis of spermidine and spermine from putrescine [40,41].

Spermidine is an aliphatic polyamine derived from putrescine, agmantine, methionine, or spermine [42]. It is a precursor to other polyamines, such as spermine and its structural isomer thermospermine. Spermidine in fish tissue can potentiate the toxic effect of histamine by inhibiting intestinal histamine-catabolic enzymes [43]. Two spermidine-related proteins were identified via LC-MS/MS (spermidine/putrescine import ATP-binding protein and S-adenosylmethionine decarboxylase proenzyme). One peptide (VDEVHDNAEAEGLIGYIR) of spermidine/putrescine import ATP-binding protein was detected via LC-ESI-MS/MS in the P. vulgaris strain. This protein is part of the ABC transporter complex PotABCD that is involved in spermidine/putrescine import [44]. In addition, one peptide (ALSFNIYDVC*YAR) of the S-adenosylmethionine decarboxylase proenzyme was detected in the *S. maltophilia* strain. This enzyme is necessary for the biosynthesis of polyamines such as spermine and spermidine from the diamine putrescine [39].

Spermine is an aliphatic polyamine derived from agmatine, methionine, or spermidine [2]. One spermine-related protein was identified via LC-ESI-MS/MS (S-adenosylmethionine decarboxylase proenzyme). A peptide (ALSFNIYDVC*YAR) of the S-adenosylmethionine decarboxylase proenzyme was detected in the *S. maltophilia* strain. In addition, spermine has been reported to modify the connections between polyamines and DNA. In fact, spermine has been reported to function as a free radical scavenger protecting DNA from oxidative stress [45]. More precisely, the higher the cationic charge, the higher the degree of DNA-protein binding enhancement; thus, spermine has been characterized as more potent than spermidine and putrescine.

Finally, further decarboxylases (e.g., phosphatidylserine decarboxylase and 4-carboxymuconolactone decarboxylase) and deaminases (e.g., 2-iminobutanoate/2-iminoopropanoate deaminase and glucosamine-6-phosphate deaminase) were identified via shotgun proteomics (Supplementary Data S1), but according to the literature, they are involved in other metabolic pathways, which was also demonstrated previously via PANTHER analysis.

### 2.5. Network Analysis

**Figure 5:**
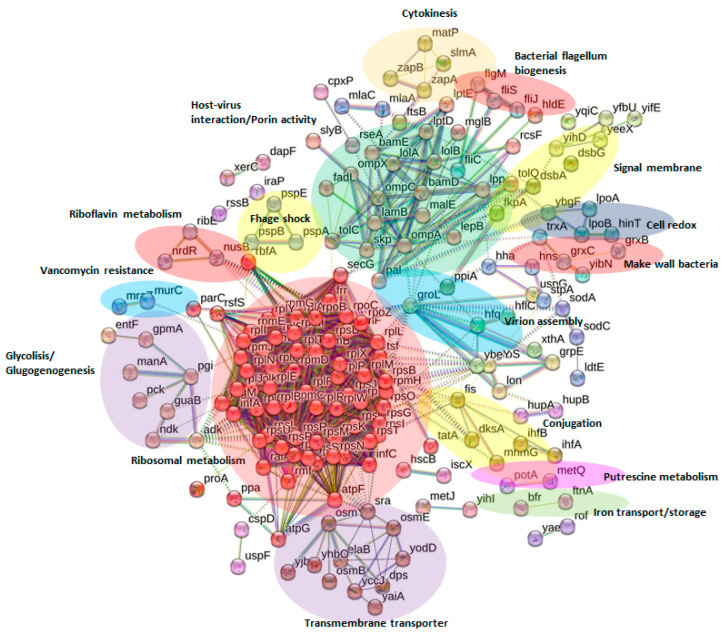
Protein interactome network for the global protein repository of biogenic-amine-producing bacterial foodborne strains using STRING v.11.5 software. High-confidence interactions (≥0.7) in STRING software were selected for this study. The final network for the global protein repository consists of 260 nodes (proteins) and 1973 edges (interactions). Nodes represent the proteins, and the interactions between proteins are represented by continuous lines when referring to direct interactions (physical) or with dotted lines when referring to indirect interactions (functional).

Network analysis was executed using STRING v.11.5 software (https://string-db.org/, accessed on 6 December 2022) [46], wherein all the proteins identified in this study were investigated and compared with the genome of the model organism *E. coli* K12 MG1655, which was the genetically closest group available in the portal (Figure 5). Every protein—protein interaction was assigned to the network in accordance with its confidence score. To reduce the occurrence of false positives and false negatives, all expected interactions were tagged as “high-confidence” (≥0.7) in the STRING program were selected for this work.

Thus, the final network for the global protein repository consisted of 260 nodes (proteins) and 1973 edges (interactions) (Figure 5). All proteins used in the network were discovered during the proteomic experiments (see the codes of the gene column in Supplementary Data S1). This protein network is the first inclusive interactomics map for relevant seafood-based, biogenic-amine foodborne strains.

Cluster networks were generated using an MCL (inflation clustering) algorithm from the STRING website, and a default value of 2 was selected for all analyses. From the cluster analysis, 42 significant clusters of interactions between nodes were obtained. Figure 5 highlights the most relevant clusters (*n* = 15) according to the abundance of nodes involved or their biological relevance. Supplementary Data S3 includes information about the 42 clusters, protein names, and descriptions of the corresponding name codes.

The most relevant subnetworks in terms of their number of nodes are involved in ribosomal metabolism (in red; 63 nodes), host-virus interaction/porin activity (in green; 22 nodes), transmembrane transport (in violet; 12 nodes), and glycolysis (in dark violet; 8 nodes).

Other subnetworks that contain fewer nodes but have great biological importance are related to bacterial flagellum biogenesis (in red; four nodes), vancomycin (an antibiotic) resistance (in blue; three nodes), and putrescine metabolism (in pink; three nodes). Further study of the aforementioned subnetworks and protein-protein interactions will be very beneficial for the development of new therapeutic treatments for bacterial dispersion, antibiotic resistance, and food intoxication via biogenic-amine-produced putrescine.

### 2.6. Virulence Factors

Many seafood-originating biogenic-amine-producing bacteria are pathogens with well-known virulence. It has been reported that *Enterobacter* bacteria are increasingly exhibiting a multidrug resistance phenotype [47]. Moreover, *K. oxytoca* can acquire antimicrobial resistance and carry multiple virulence genes, such as capsular polysaccharides and fimbriae [48]. The virulence of other species analyzed in this study, such as *H. alvei* [49], *M. morganii* [50], *P. vulgaris* [51], *S. maltophilia* [52], and *R. planticola* [53], has been previously reported.

A total of 556 peptides belonging to virulence factors (nonredundant peptides) were identified in this study. They included toxins, polypeptides involved in antibiotic resistance, and proteins related to cell colonization and immune evasion. The 556 virulent peptides (Supplementary Data S4) are displayed in groups in accordance with the principal roles in which they are involved (e.g., toxin generation/transport, colonization and immune evasion factors, antimicrobial compounds, other tolerance proteins that play a role in resistance to toxic substances, etc.). In addition, the main proteins of the identified virulence factors are displayed in Table 3.

In this study, several peptides involved in antimicrobial resistance or the production/transport of toxic substances were identified (Supplementary Data S4). Ten of the proteins characterized were associated with antibiotic resistance, and 91 peptides were related to other tolerances. Four peptides were identified as penicillin-binding proteins. Peptides associated with acriflavine and methicillin resistance and belonging to the TetR family of regulators (TFRs) were determined. TetR proteins regulate antibiotic and quorum-sensing processes as well as antibiotic resistance. In addition, two peptides of the GCN-2-related N-acetyl transferase (GNAT) family of acetyltransferases, which provide antibiotic resistance [54], were also identified. Peptides of proteins involved in other bacterial tolerances (e.g., thermotolerance and osmotolerance) were also identified. Accordingly, this work has identified many peptides that belong to groups of peptides of bacterial general stress response proteins, heat shock proteins, and cold-shock-like proteins (CSPs), among others [55,56].

A total of 20 peptides corresponding to proteins that are involved in bacterial toxicity were identified. These peptides include ecotin, lipoprotein toxin enterocidin B, antitoxin ParD, and addition module toxin GnSA/GnsB. As an example of some of the roles these peptides play, ecotin is an inhibitor of multiple complement-dependent processes found in bacteria [57].

In this study, 349 peptides involved in colonization and immune evasion were identified. Bacterial internalization into the host is facilitated by these proteins, resulting in subsequent infection and propagation. Transcriptional regulators involved in the control of virulence factors were also found for the analyzed strains, including two peptides identified as LysR and SlyA [58,59]. LysR regulates virulence factors, such as extracellular polysaccharides, toxins, and bacteriocins. Fimbria are located on the surfaces of bacteria; they are involved in adherence to target cells and biofilm formation [60]. Lysis proteins belonging to the LysM domain were identified; this domain was identified in enzymes involved in bacterial cell wall degradation [61]. Additionally, several peptides of peptidases and proteases were identified. This includes members of the Lon protease family and subtilisin, among others. Different peptides of the Superoxide dismutase enzyme (SOD) were identified. SOD is a metalloenzyme that defends against reactive oxygen species produced by neutrophils and macrophages [62]. The presence of open channels facilitates passive penetration though the outer membrane. We have identified several porins or outer-membrane proteins (OMPs), such as the porins OmpA, OmpX, and OmpC; substrate-specific porins, such as maltoporin, which is also called LamB; and TonB-dependent receptors, such as FhuA [47]. In addition, many peptides were determined to be other virulence factors, such as VacJ family lipoprotein VacJ (virulence-associated chromosome locus J); the chaperone protein Skp, which assists in the folding and insertion of many OMPs [63]; and the Osmy chaperone.

In this study, four peptides of antibacterial proteins were identified, including one peptide that belongs to a bacteriocin and the remaining three to a colicin-like protein. Colicins are antimicrobial proteins typically produced by *E. coli* that degrade internal cellular elements [64].

ABC transporters, like many other bacterial transporters, are involved in resistance or tolerance and bacterial propagation during infection [65]. We identified different ABC transporters related to virulence (Table 3).

Furthermore, sixteen peptides of alternative virulent factors were identified, such as proteins related to mobile genetic elements’ transposases, recombinases, plasmids, and viral DNA fragments, which are considered the major mechanism for acquiring antibiotic resistance. Moreover, pilus conforms to a typical method of horizontal transfer between bacteria, which is another mechanism of obtaining virulence determinants [66]. We identified 43 peptides of phage proteins, such as bacteriophage CI repressor and capsid scaffolding protein, but mainly phage shock proteins. Finally, we identified bacterial proteins determined in the UniProt database in different phage strains (*Klebsiella* phage vB_KpM_FBKp24, *Klebsiella* phage vB_KppS-Storm, *Stenotrophomonas* phage BUCT608, and *Stenotrophomonas* phage).

### 2.7. Potential Species-Specific Peptide Biomarkers

To select potential peptide biomarkers for the 15 different biogenic-amine-producing bacterial strains, we implemented a massive comparison of the proteomics data with respect to the proteins and peptides included in databases. The suitable peptides that were identified via LC-ESI-MS/MS in only one specific species were verified in terms of their specificity and sequence homology using the BLASTp algorithm [67] (Supplementary Data S5). Table 4 summarizes the analysis of the 77 species-specific tryptic peptide biomarkers belonging to 64 different proteins that were suggested for the unequivocal identification of the different seafood-originating biogenic-amine-producing bacteria of 10 different species (*E. aerogenes*, *E. cloacae*, *H. alvei*, *K. oxytoca*, *M. morganii*, *P. mirabilis*, *P. penneri*, *P. vulgaris*, *R. planticola*, and *S. maltophilia*).

All the peptides included herein have been proposed as potential biomarkers for the first time and will be very convenient for further studies using targeted proteomics approaches to identify the different seafood-originating biogenic-amine-producing bacteria in foodstuffs.

## 3. Materials and Methods

### 3.1. Bacterial Strains

A total of 15 different seafood-originating biogenic-amine-producing bacteria were included in this work (Table 1). Strains were previously studied via MALDI-TOF-MS and 16S rRNA sequencing [10,68]. All bacterial strains were activated in brain–heart infusion (BHI) and incubated in vials at 31 °C for 24 h. Then, strain cultures were expanded on plate count agar (PCA) at 31 °C for 24 h. Samples were prepared in triplicate.

### 3.2. Protein Extraction

Protein extracts were obtained as described by Carrera et al. (2017) [24,69,70]. Concisely, the biomass of bacterial cells was mixed with a solution of 1% trifluoracetic acid/50% acetonitrile. After several extractions with glass beads conducted for 10 min at 4 °C, the supernatants were centrifuged for 10 min at 40,000× *g* (J221-M centrifuge, Beckman, Brea, CA, USA). The supernatant was then solubilized with lysis buffer containing 60 mM Tris-HCl pH 7.5, 1% lauryl maltoside, 5 mM phenylmethanesulfonylfluoride (PMSF), and 1% dithiothreitol (DTT). The solution was transferred to a new vial, and the quantity of protein was revealed via the bicinchoninic acid method (Sigma Chemical Co., St. Louis, MO, USA). This method was chosen because a similar procedure has been applied previously for protein extraction via MALDI-TOF MS analysis [68].

### 3.3. Peptide Sample Preparation

Proteins were digested with trypsin, as described previously [71]. A total of 100 μg of protein extracts was dried under vacuum and solubilized in 25 μL of 8 M urea in 25 mM of ammonium bicarbonate at pH 8.0. After 5 min of sonication, DTT was added at a final concentration of 10 mM and incubated at 37 °C for 1 h. Then, iodoacetamide was supplemented at a final concentration of 50 mM and incubated at room temperature in darkness for 1 h. Next, the sample was diluted four times to a final concentration of 2 M urea with 25 mM ammonium bicarbonate (pH 8.0) and subjected to digestion with trypsin (ratio 1:100) (Promega, Wisconsin, WI, USA) at 37 °C overnight.

### 3.4. Shotgun LC-ESI-MS/MS Analysis in a LTQ-Orbitrap Instrument

Peptides were acidified with 5% formic acid (FA) until attaining pH 2, cleaned on a C18 MicroSpinTM column (The Nest Group, Southborough, MA, USA), and analyzed via LC-MS/MS using a Proxeon EASY-nLC II LC machine (Thermo Scientific, San Jose, CA, USA) coupled with an LTQ-Orbitrap XL (Thermo Fisher Scientific). Separation of peptides (2 µg) was implemented on an RP column (EASY-Spray column, 50 cm × 75 µm ID, PepMap C18, 2 µm particles, 100 Å pore size, Thermo Fisher Scientific) with a 10 mm precolumn (Accucore XL C18, Thermo Scientific) containing 0.1% FA in Milli-Q water and 98% ACN and 0.1% FA as mobile phases A and B, respectively. A 240 min linear gradient from 5 to 35% B at a flow rate of 300 nL/min was used. A capillary temperature of 230 °C and spray voltage of 1.95 kV were used for ionization. Peptides were analyzed from 400 to 1600 amu (1 µscan) in positive mode, followed by 10 data-dependent CID MS/MS scans (1 µscans) using an isolation width of 3 amu and a normalized collision energy of 35%. Fragmented masses were set in dynamic exclusion for 30 s after the second fragmentation event. Unassigned charged ions were omitted from MS/MS analysis.

### 3.5. LC-ESI-MS/MS Data Processing

MS/MS spectra were identified using SEQUEST-HT (Proteome Discoverer 2.4 package, Thermo Fisher Scientific) and compared to the Proteobacteria UniProt/TrEMBL database (with 2,627,375 protein sequence entries dating from August 2022). MS/MS spectra were analyzed using fully tryptic cleavage constraints, and up to two missed cleavage sites were permissible. Windows for tolerance were set at 10 ppm for precursor ions and 0.06 Da for MS/MS fragment ions. The variable modifications permitted were methionine oxidation (Mox), carbamidomethylation of Cys (C*), and acetylation of the N-terminus of a protein (N-Acyl).

The results were subjected to statistical analysis to determine the false discovery rate (FDR) regarding peptides using a decoy database and the Percolator algorithm included in the Proteome Discoverer 2.4 program [72]. The FDR was kept below 1% for further analysis. The MS/MS proteomics data have been deposited to the ProteomeXchange Consortium via PRIDE with the dataset identifier PXD039320.

To determine relative protein abundance for each strain, a label-free quantification (LFQ) method was used by applying the Minora Feature Detector node and the ANOVA (individual proteins) method included in the Proteome Discover 2.4 software (Thermo Fisher Scientific). Peak areas of ion features from the same peptide for different charge forms were combined into one value.

### 3.6. Euclidean Hierarchical Clustering

The function heatmap.2 of the statistical package R (version (v) 4.1.1) (http://www.r-project.org, accessed on 25 January 2023) was used to achieve the Euclidean hierarchical clustering of the data. The Ggplots v.4.1.1 package, the Euclidean distance metric, and the complete linkage for the agglomeration method were used as constraints.

### 3.7. Functional Analysis: Gene Ontology (GO) and Pathways Analysis

The nonredundant protein IDs (column “Gene name” in Supplemental Data S1) were submitted to the PANTHER software (http://www.pantherdb.org/, accessed on 30 November 2022) for grouping established based on the following main types of interpretations: molecular function, biological process, and protein class. The statistical significance was also provided as a percentage. For this procedure, all orthologous gene ID entries were included as a reference set. The pathway analysis data were clustered, thus providing an approximation of the statistical significance of over- or underrepresentation according to the GO descriptors of the proteins in the proteome.

### 3.8. Network Analysis

Protein network was developed by incorporating the orthologous gene IDs into the STRING program (v.11.5) (http://string-db.org/, accessed on 6 December 2022) [46]. STRING is an enormous database of known and predicted protein interactions. Proteins are denoted with nodes, and interactions are represented as continuous lines. All edges were reinforced by at least one reference from the literature or from canonical information deposited in the STRING dataset. The confidence score was set at ≥0.7 (high confidence). MCL algorithm included on the STRING website was used to generate cluster networks, and a default value of 2 was assigned for all analyses.

### 3.9. Virulence Factors

Virulence Factor of Pathogenic Bacteria Database (VFDB) (http://www.mgc.ac.cn/VFs/, accessed on 13 December 2022) was used to characterize virulence factors. Additionally, we prolonged the analysis to include virulence factors that are contained in several scientific publications [47,48,49,50,51,52,53].

### 3.10. Selection of Potential Peptide Biomarkers

BLASTp algorithm applied to each identified peptide by LC-MS/MS was used to determine homologies and exclusiveness with respect to protein sequences recorded in the NCBI database [67].

## 4. Conclusions

This article presents the first shotgun proteomics study of 15 different foodborne strains of biogenic-amine-producing bacteria. By means of a rapid and easy procedure for preparing proteins, the results were used to differentiate several protein datasets, which, in turn, were used to determine relevant functional pathways and differentiate strains into different Euclidean hierarchical clusters. Additionally, a predicted protein-protein interaction network for foodborne biogenic-amine-producing bacteria was created. Most proteins were classified under pathways and networks related to energy, putrescine metabolism, and host-virus interactions. Additionally, 556 different virulence factors were identified. Most of these factors corresponded to functions/roles such as toxins, antimicrobial compound production, antimicrobial resistance, additional resistances and tolerances, host colonization and immune evasion, ABC transporters, phage proteins, and alternative virulence factors and proteins involved in horizontal transfer. Finally, 77 prospective species-specific peptide biomarkers corresponding to 64 different proteins were screened to identify unique potential peptide biomarkers for 10 biogenic-amine-producing bacterial species. To date, these results constitute the largest dataset of peptides and proteins from foodborne biogenic-amine-producing bacterial species strains. This repository provides data that can be used in further studies to develop new therapeutic treatments for biogenic-amine-producing bacterial species with respect to food intoxication and for the tracking of microbial sources in foodstuffs.

## Figures and Tables

**Table 1:** The biogenic amine-producing bacterial strains considered in this study.

Sample	Bacterial Strain	Code	Source	GenBank
H1	*Enterobacter aerogenes*	EbAe1	ATCC 13048	FJ971882
H2	*Enterobacter cloacae*	EbCl1	ATCC 13047	FJ971883
H3	*Hafnia alvei*	HaAl2	ATCC 9760	FJ971884
H4	*Klebsiella oxytoca*	KlOx1	ATCC 13182	FJ971867
H5	*Morganella morganii*	MoMo1	BM 65	FJ971858
H6	*Morganella morganii*	MoMo2	ATCC 8076	FJ971868
H7	*Proteus mirabilis*	PrMi1	ATCC 14153	FJ971887
H8	*Proteus penneri*	PrPe1	ATCC 33519	FJ971869
H9	*Proteus vulgaris*	PrVu1	ATCC 9484	FJ971888
H10	*Proteus vulgaris*	Sard1	Sardine	JN630885
H11	*Proteus vulgaris*	Sard2	Sardine	JN630886
H12	*Raoultella planticola*	RaPl2	ATCC 33531	FJ971885
H13	*Stenotrophomonas maltophilia*	25MC6	Albacore tuna	FJ971861
H14	*Stenotrophomonas maltophilia*	5PC6	Albacore tuna	FJ971863
H15	*Stenotrophomonas maltophilia*	StMa2	15MF	FJ971862

**Table 2:** Biogenic amine-related proteins and peptides detected via LC-ESI-MS/MS for the corresponding strains.

Biogenic Amine	Precursor	Proteins Identified by LC-ESI-MS/MS	Peptides	Sample	Bacterial Strain
Agmatine	Arginine	Arginine ABC transporter substrate-binding protein	IDAVFGDTAVVTEWLK	H4	*K. oxytoca*
		Lysine-arginine-ornithine-binding periplasmic protein	C*TWVGSDFDSLIPSLK	H4	*K. oxytoca*
		“	IGTDATYAPFSSK	H4	*K. oxytoca*
Cadaverine	Lysine	Lysine-arginine-ornithine-binding periplasmic protein	C*TWVGSDFDSLIPSLK	H4	*K. oxytoca*
		“	IGTDATYAPFSSK	H4	*K. oxytoca*
Histamine	Histidine	Histidine kinase	IDSEDLPHVRASVAR	H6	*M. morganii*
		“	LAM*NLRTRLFLSISALITVALLGLLLGLVSVM*QM*AGSQEILIR	H13	*S. maltophilia*
		“	M*IAEAANADSKQAQR	H4, H12	*K. oxytoca, R. planticola*
		“	TIDQINQQKIQLEQEIADRK	H8, H9	*P. penneri, P. vulgaris*
		“	GEADATLDSEVSAWRAVAR	H11	*P. vulgaris*
		“	LSSELWNC*KIDPTQAEM*AM*INILANAR	H7	*P. mirabilis*
		“	SEASENTVDLIVEDEGSGIPK	H7	*P. mirabilis*
		“	NEEARDNLKLISELTAR	H11	*P. vulgaris*
		“	RYAYSEQLGDLLQR	H15	*S. maltophilia*
		Histidine phosphatase	HAQASEYGSALFVAVGQAKQVK	H3	*H. alvei*
		Histidine-binding periplasmic protein	IGVLQGTTQETYGNEHWAPK	H4	*K. oxytoca*
		Histidine triad nucleotide-binding protein	EIPSDIVYQDELVTAFR	H4	*K. oxytoca*
		“	IAEQEGIAEDGYR	H2	*E. cloacae*
		Histidine ammonia-lyase	LAAM*QQALGAQIAAVEEDR	H2	*E. cloacae*
				H6	*M. morganii*
Putrescine	Arginine	Lysine-arginine-ornithine-binding periplasmic protein	C*TWVGSDFDSLIPSLK	H4	*K. oxytoca*
		“	IGTDATYAPFSSK	H4	*K. oxytoca*
	Glutamine	Glutamine ABC transporter periplasmic protein	AVGDSIEAQQYGIAFPK	H4	*K. oxytoca*
		Glutamine-fructose-6-phosphate aminotransferase	IDAAQEAELIKALFEAPR	H4	*K. oxytoca*
		N-acetylglutaminylglutamine amidotransferase	SGANAAVDKALRLDSTVM*LVDDPVK	H3	*H. alvei*
	Methionine	Type 1 glutamine amidotransferase domain-containing protein	IFRTLALM*LLVTSATAFAASK	H7	*P. mirabilis*
		L-glutamine-binding protein	ADAVIHDTPNILYFIK	H4	*K. oxytoca*
	Ornithine	“	AVGDSLEAQQYGIAFPK	H3	*H. alvei*
		S-adenosylmethionine decarboxylase proenzyme	ALSFNIYDVC*YAR	H15	*S. maltophilia*
Spermidine	Agmatine	Spermidine/putrescine import ATP-binding protein	VDEVHDNAEAEGLIGYIR	H9	*P. vulgaris*
	Methionine	S-adenosylmethionine decarboxylase proenzyme	ALSFNIYDVC*YAR	H15	*S. maltophilia*
	Putrescine				
	Spermine				
Spermine	Agmatine				
	Methionine	S-adenosylmethionine decarboxylase proenzyme	ALSFNIYDVC*YAR	H15	*S. maltophilia*
	Spermidine				

C* (carbamidomethyl cysteine), M*(methionine oxidation).

**Table 3:** Proteins corresponding to bacterial resistance to antibiotics, antimicrobial-related proteins, and other virulence factors identified in the strains analyzed.

Funtion	Protein	Sample
**Toxins**	Entericidin A/B family lipoprotein	H1, H2, H3, H4
	Addiction module toxin, GnsA/GnsB family	H12
	Antitoxin ParD	H15, H13
	Ecotin	H1, H3, H4
**Antimicrobial compounds production**	Bacteriocin immunity protein	H9
	Colicin immunity protein/pyocin immunity protein	H1
**Antimicrobial resistance**	Penicillin-binding protein activator LpoB	H4
	TetR family transcriptional regulator	H5, H15
	Acriflavine resistance protein B	H61
	Methicillin resistance protein	H15
	GNAT family N-acetyltransferase	H1, H5
**Additional resistances and tolerances**	Acid stress chaperone HdeB	H5, H6
	Cold shock protein CspA	H4, H14
	Cold shock protein CspC	H4
	Cold shock protein CspD	H14, H6, H8
	Cold shock protein CspE	H4
	Copper resistance protein	H5, H6
	Envelope stress response membrane protein PspB	H1, H3, H5, H8, H9
	General stress protein	H4, H13, H15
	Heat shock survival AAA family ATPase ClpK	H2, H4
	Stress response protein ElaB	H13
	Stress response translation initiation inhibitor YciH	H5
	Stress-induced bacterial acidophilic repeat motif	H2
	Universal stress protein	H2, H3, H4, H12
	YdeI family stress tolerance OB fold protein	H2
	CsbD family protein	H2, H3, H9, H15
	Peroxide/acid resistance protein YodD	H2
	Putative ‘Cold-shock’ DNA-binding domain protein	H15
	L,D-transpeptidase YnhG	H4
	Protein sufA	H5
	Spy/CpxP family protein refolding chaperone	H4
**Host colonization and immune evasion**	Beta-aspartyl-peptidase	H9
	Chaperone protein Skp	H1, H2, H3, H4, H5, H7, H8, H9, H12
	Chemotaxis protein CheY	H13
	Cytosol nonspecific dipeptidase	H2
	Filamentous hemagglutinin	H2
	Fimbrial protein FimV	H15
	Type 1 fimbrial protein	H8
	Flagellar biosynthesis protein FliC	H14
	Flagellar hook protein FlgE	H13
	Flagellar secretion chaperone FliS	H15
	Flagellin	H8, H10, H11, H13, H14, H15
	Hemagluttinin	H1
	Hemolysin expression modulator Hha	H3, H7
	Inhibitor of vertebrate lysozyme	H3
	Isoaspartyl peptidase/L-asparaginase	H7, H11, H13, H15
	Lipoprotein involved in copper homeostasis and adhesion	H4
	Lon protease	H1
	LysM	H2, H4, H13, H15
	LysR family transcriptional regulator	H12
	Maltoporin	H4
	Ferrichrome porin FhuA	H4
	MipA/OmpV family protein	H4
	Molecular chaperone OsmY	H2, H3, H4
	OmpA	H1, H2, H3, H4, H8, H13
	OmpC	H4, H10
	OmpD	H4
	OmpF	H4
	OmpK36	H4
	Omptin family outer membrane protease	H4
	OmpX	H4
	Phosphate-selective porin OprO/OprP	H15
	OsmC family peroxiredoxin	H4
	Outer membrane lipoprotein RcsF	H4
	Peptidase S74	H15
	Peptidase S8 and S53 subtilisin kexin sedolisin	H13
	Periplasmic serine endoprotease DegP-like	H15
	IAP aminopeptidase MEROPS family M28C	H1
	Membrane-bound metallopeptidase	H9
	Metalloprotease	H4
	Alkaline serine protease	H14
	Signal peptidase I	H12
	Superoxide dismutase	H4, H7, H9, H15, H13, H2
	Tautomerase PptA	H2
	Tautomerase ydcE	H12
	TonB	H4, H14, H15
	Transcriptional regulator SlyA	H1, H2, H3, H4, H12
	Twitching motility protein PilH	H14
	Type I restriction enzyme endonuclease	H4
	Type VI secretion protein	H15
	VacJ family lipoprotein	H15
**ABC transporters**	Amino acid ABC transporter	H2
	Arginine ABC transporter substrate-binding protein	H4
	Glutamine ABC transporter periplasmic protein	H3, H4
	Manganese ABC transporter	H4
	Iron ABC transporter	H4
	Oligopeptide ABC transporter	H5, H6
	Putative ABC-type sugar transport system	H1
	Ribose ABC transporter substrate-binding protein RbsB	H4
	Xylose ABC transporter, periplasmic xylose-binding protein XylF	H2
**Phage proteins**	Presumed capsid scaffolding protein (GpO)	H10
	Bacteriophage CI repressor	H1
	Beta_helix domain-containing protein (*Klebsiella* phage vB_KpM_FBKp24)	H7
	Phage portal protein, HK97 family	H5
	Phage shock protein PspA	H1, H2, H3, H4, H5, H6, H7, H9, H11, H12
	Uncharacterized protein (*Stenotrophomonas* phage BUCT608)	H12
	Terminase (Klebsiella phage vB_KppS-Storm)	H11
	Uncharacterized protein (*Stenotrophomonas* phage Marzo)	H11
**Alternative virulence factors and proteins involved in horizontal transfer**	Pilus assembly protein, pilin FimA	H5
	IS3 family transposase	H5
	Major type 1 subunit fimbrin (Pilin)	H12
	Plasmid stability protein	H3
	Plasmid-related protein	H7
	Rop family plasmid primer RNA-binding protein	H5
	Transposase	H15
	Tyrosine recombinase XerC	H5
	MobC family plasmid mobilization relaxosome protein	H5

**Table 4:** Potential species-specific tryptic peptide biomarkers of seafood-originating biogenic-amine-producing bacteria. Specificity was determined after similarity search using BLASTp.

Protein	Peptide	Sample	Specific by Blastp
DUF883 domain-containing protein	NLADTLEEVLNSSTDKSKEELGK	H1	*E. aerogenes*
Uncharacterized protein	LLQLALAAIDSADEAGVSHEDIDNQHTEEEASPLTPK	H1	*E. aerogenes*
Hemagluttinin domain-containing protein	SVAQNAAAITDTR	H1	*E. aerogenes*
Anti-adapter protein IraP	LIQDIETAMEQVKPGPLVDDRDTQLLQQYIK	H1	*E. aerogenes*
Cell division protein ZapB	NTTLAQEVQSAQHGREELERENSQLR	H1	*E. aerogenes*
Der GTPase-activating protein YihI	KPIPLGVTESTPAVK	H1	*E. aerogenes*
DUF1471 domain-containing protein	AREEGAKGFVVNSAGGDNHMYGTATIYK	H2	*E. cloacae*
DUF2511 domain-containing protein	SSGQPISVIQIDDPSSPGQK	H2	*E. cloacae*
Uncharacterized protein	ESGFEGELTDLSDDILIYHLK	H2	*E. cloacae*
Universal stress protein UspF	M*FNSILVPVDISESR	H2	*E. cloacae*
DNA-binding protein	IKDNNAEYVEPLDMLAELC*EDNKLLAAELR	H3	*Hafnia alvei*
ATP synthase subunit b	KAQIIDEAKVEAEQER	H3	*Hafnia alvei*
DUF883_C domain-containing protein	GVANEAAGQVEESYGEATNSHQHRLEGQAR	H3	*Hafnia alvei*
Exodeoxyribonuclease 7 small subunit	APAAPSFEQALSELEQIVTHLESGELPLEDALNEFER	H3	*Hafnia alvei*
ATP synthase subunit b	AQIIDEAKVEAEQERNK	H3	*Hafnia alvei*
Cell division protein ZapB	ESLVRENEQLKEEQTAWQER	H3	*Hafnia alvei*
50S ribosomal protein L10	IVEGTPFEC*LKDTFVGPTLVAFSMEHPGAAAR	H3	*Hafnia alvei*
Uncharacterized protein	RKLSPAEELALGK	H3	*Hafnia alvei*
Major outer membrane lipoprotein Lpp	VDQLSNDVNAM*RADVQTAKDDAAR	H3	*Hafnia alvei*
DUF1471 domain-containing protein	IGDVSAEVRDGTM*DDIVK	H3	*Hafnia alvei*
Uncharacterized protein	ESDAEREEKTFTWKPSAVR	H3	*Hafnia alvei*
Cell division protein ZapB	ENEQLKEEQTAWQER	H3	*Hafnia alvei*
Membrane protein	NGVPESGFTLDVVPNDQADASGGQVVGHC*ENDTQK	H3	*Hafnia alvei*
Chaperone protein Skp	ATELQGQERDLQSK	H3	*Hafnia alvei*
Outer membrane lipoprotein SlyB	AVQIQGGDESNAIGAIGGAVLGGFLGNTIGGGTGR	H4	*K. oxytoca*
Putative porin	NYVEANGGISWTPLTPLTIK	H4	*K. oxytoca*
TolC-like protein	QAGIQDVTYQTDQQTLILNTATAYFK	H4	*K. oxytoca*
Maltoporin	SSESGGSGTFADRDQFGNR	H4	*K. oxytoca*
Glutamate/aspartate ABC transporter substrate-binding protein	LIPVTSQNRIPLLQNGTFDFEC*GSTTNNLAR	H6	*M. morganii*
Integration host factor subunit alpha	AEM*SENLSEKLDLSKR	H5	*M. morganii*
FAD assembly factor SdhE	GRPDDEALYQIIR	H5	*M. morganii*
Cell division protein ZapB	VQQALDTITLLQM*EIEELKEKNDALNQEVQGAR	H7	*P. mirabilis*
Outer membrane protein assembly factor BamE	VRQETLTLTFDNNGILTK	H7	*P. mirabilis*
DUF2594 family protein	AISEVADEQQAETFRNTLNQIK	H7	*P. mirabilis*
DNA-binding protein	IIADFLGVAPSEIWPSRYFHPETGELLER	H7	*P. mirabilis*
Inner membrane protein YhcB	SSNELMPDMPEQENPFNYR	H8	*P. penneri*
DUF1043 family protein	SSNELM*PDM*PAQDNPFNYR	H9	*P. vulgaris*
Lipoprotein	QQAQETENQALDKADQLTDQAK	H9	*P. vulgaris*
Arsenate reductase	SLELADPQLSEDALIQAIVDNPK	H12	*R. planticola*
Uncharacterized protein	SEHAAQGKSDSVGSQVSEGAQKTWNK	H12	*R. planticola*
Uncharacterized protein	EAEQLDNDKNFYYQEAK	H12	*R. planticola*
Exported protein	VGTISSTGQTAPGDARAELLK	H12	*R. planticola*
Uncharacterized protein	TPLSDTDFANKILASQANQEYVR	H12	*R. planticola*
DUF1311 domain-containing protein	SRDGELDTALYDDSQPGNLQGELNDVMR	H15	*S. maltophilia*
Type VI secretion protein	NAPAADTQNFYNAPAPR	H15	*S. maltophilia*
Uncharacterized protein	VLAAGGTAAQALAASQAAAR	H15	*S. maltophilia*
Uncharacterized protein	GSTTVAGQDISLNQDFK	H15	*S. maltophilia*
Uncharacterized protein	DQKDLPHPDAEAQRPDPVSPLQAK	H15	*S. maltophilia*
DNA-binding protein	SLIAQAEKQQSK	H15	*S. maltophilia*
Uncharacterized protein	AQSTQDLGLHTSC*R	H15	*S. maltophilia*
Uncharacterized protein	TYFDYSEEQPFIR	H15	*S. maltophilia*
30S ribosomal protein S16	VGFYNPVAQGGEK	H15	*S. maltophilia*
Cu(I)-responsive transcriptional regulator	LGEDDQTPVPVDTAK	H15	*S. maltophilia*
Antitoxin ParD	QLIAEGLASGPSAPLAPDHFDKLR	H15	*S. maltophilia*
DUF3606 domain-containing protein	AAVAEVGPTAAAVR	H15	*S. maltophilia*
TonB-dependent receptor	NASSGPGAVVLSPTHPDNPIPGQASR	H15	*S. maltophilia*
RidA family protein	AFDNLKAVAEAAGGSLDQVVR	H13	*S. maltophilia*
LysM peptidoglycan-binding domain-containing protein	KADFSGVSGSVDSTAEQVPK	H13	*S. maltophilia*
Uncharacterized protein	SANAAATAAQEAADAAAAK	H13	*S. maltophilia*
DUF4124 domain-containing protein	ANLALLDGGGQVMQDTDGDGKADTPLAPEQR	H13	*S. maltophilia*
LysM peptidoglycan-binding domain-containing protein	ADFSGVSGSVDSTAEQVPK	H13	*S. maltophilia*
DUF4124 domain-containing protein	SPQAAASAETPAAPVPEQC*STAR	H13	*S. maltophilia*
Flagellin	FTSTIANLNTNSENLSAAR	H13	*S. maltophilia*
Uncharacterized protein	DAADKTAAASEQAAADTQQALDKAADATANAADQAK	H13	*S. maltophilia*
Attachment protein	LLGDIAKDLTNAPLEDIQK	H13	*S. maltophilia*
Antitoxin ParD	QLIAEGLASGPAVPVTAATFER	H13	*S. maltophilia*
Uncharacterized protein	TQADAILADSAANEYDKSLAAQLASQAAYQTDDTPAAVAYLK	H13	*S. maltophilia*
Excinuclease ATPase subunit	VEQSLQELISSQAAK	H13	*S. maltophilia*
Uncharacterized conserved protein, DUF2147	SIVEISQAANGTLTGK	H13	*S. maltophilia*
50S ribosomal protein L31 type B	STM*GTKETIQWEDGNEYPLVK	H13	*S. maltophilia*
LysM peptidoglycan-binding domain-containing protein	ADFSGVSASVDSTADVVSGGTYTVQKGDSLSK	H13	*S. maltophilia*
DUF3613 domain-containing protein	SFEHEIPDFFEADVAK	H13	*S. maltophilia*
Poly(Hydroxyalkanoate) granule-associated protein	LHVPTADEVTALEARIDALQAR	H13	*S. maltophilia*
Heme exporter protein D	AVKQDAAAPLSTELER	H13	*S. maltophilia*
RNA polymerase-binding transcription factor DksA	TDEATGRPILPTGYKPGSEEEYM*SPLQQEYFR	H13	*S. maltophilia*
Transcriptional regulator	LEALDALLPSDSPNPIDLLER	H13	*S. maltophilia*
CsbD-like protein	IQKGVGEVQSDVGKAR	H13	*S. maltophilia*

C* (carbamidomethyl cysteine); M* (methionine oxidation).

## Data Availability

The MS/MS proteomics data have been deposited to the ProteomeXchange Consortium via PRIDE with the dataset identifier PXD039320.

## Supplementary Materials

The following supporting information can be downloaded at: https://www.mdpi.com/article/10.3390/ijms24097704/s1.
